# The Prevalence of *Bartonella* Bacteria in Cattle Lice Collected from Three Provinces of Thailand

**DOI:** 10.3390/insects10060152

**Published:** 2019-05-28

**Authors:** Chulaluk Promrangsee, Pathamet Khositharattanakool, Puckavadee Somwang, Sakone Sunantaraporn, Atchara Phumee, Kanok Preativatanyou, Apiwat Tawatsin, Narisa Brownell, Padet Siriyasatien

**Affiliations:** 1 Medical Parasitology Program, Department of Parasitology, Faculty of Medicine, Chulalongkorn University, Bangkok 10330, Thailand; famezaflamingoclub@hotmail.com; 2 School of Medicine, Mae Fah Luang University, Chiang Rai 57100, Thailand; pathamet.kho@mfu.ac.th (P.K.); puckavadee.som@mfu.ac.th (P.S.); 3 Medical Science Program, Faculty of Medicine, Chulalongkorn University, Bangkok 10330, Thailand; narmspace_open@hotmail.com; 4 Vector Biology and Vector Borne Disease Research Unit, Department of Parasitology, Faculty of Medicine, Chulalongkorn University, Bangkok 10330, Thailand; amphumee@gmail.com (A.P.); junior_science@windowslive.com (K.P.); natnarisa@gmail.com (N.B.); 5 Thai Red Cross Emerging Infectious Diseases-Health Science Centre, World Health Organization Collaborating Centre for Research and Training on Viral Zoonoses, Chulalongkorn Hospital, Bangkok 10330, Thailand; 6 Department of Medical Sciences, Ministry of Public Health, National Institute of Health, Nonthaburi 11000, Thailand; atawatsin@gmail.com

**Keywords:** cattle louse, *Bartonella* spp., *18S rRNA*, *gltA*, *rpoB*, Thailand

## Abstract

Cattle lice are obligatory blood-sucking parasites, which is the cause of animal health problems worldwide. Recently, several studies have revealed that pathogenic bacteria could be found in cattle lice, and it can act as a potential vector for transmitting louse-borne diseases. However, the cattle lice and their pathogenic bacteria in Thailand have never been evaluated. In the present study, we aim to determine the presence of bacterial pathogens in cattle lice collected from three localities of Thailand. Total genomic DNA was extracted from 109 cattle louse samples and the Polymerase Chain Reaction (PCR) of *18S rRNA* was developed to identify the cattle louse. Moreover, PCR was used for screening *Bartonella* spp., *Acinetobacter* spp., and *Rickettsia* spp. in cattle louse samples. The positive PCR products were cloned and sequenced. The phylogenetic tree based on the partial *18S rRNA* sequences demonstrated that cattle lice species in this study are classified into two groups according to reference sequences; *Haematopinus quadripertusus* and *Haematopinus* spp. closely related to *H. tuberculatus*. The pathogen detection revealed that *Bartonella* spp. DNA of *gltA* and *rpoB* were detected in 25 of 109 samples (22.93%) both egg and adult stages, whereas *Acinetobacter* spp. and *Rickettsia* spp. were not detected in all cattle lice DNA samples. The *gltA* and *rpoB* sequences showed that the *Bartonella* spp. DNA was found in both *H. quadripertusus* and *Haematopinus* spp. closely related to *H. tuberculatus*. This study is the first report of the *Bartonella* spp. detected in cattle lice from Thailand. The finding obtained from this study could be used to determine whether the cattle lice can serve as a potential vector to transmit these pathogenic bacteria among cattle and may affect animal to human health.

## 1. Introduction

Cattle lice are obligatory blood-sucking ectoparasites, which is an obstacle in the health and product performance of livestock [1]. Two major families of lice have been found on cattle; Haematopinidae family, including *Haematopinus eurysternus*, *H. quadripertusus*, *H. tuberculatus* and Linognatidae family; *Linognathus vituli* and *Solenopotes capillatus* [2]. Especially, the cattle tail blood-sucking louse, *H. quadripertusus*, is commonly found to infest on the cattle tail hair and is widespread in tropical and subtropical regions [3]. Lice infestation are a common cause of animal health and they can be responsible for economic losses by inducing pathophysiological changes in their hosts, including weight loss, skin infections and damage, loss of wool or hair due to scratching, and can cause mild to severe anemia [4]. In Thailand, the domestic cattle and buffaloes lice were reported in 18 provinces of central, eastern, northeastern and southern regions, which were identified as *H. eurysternus*, *H. quadripertusus* and *L. vituli* [5]. *S. capillatus* was first reported in Tak Province, Thailand by Changbunjong et al. (2009) [6]. The relevant information on pathogens in association with cattle lice, their hosts, geographic distribution, seasonality, and association with human or veterinary diseases is limited. Currently, several reports suggested that cattle lice are potentially vector of bacterial pathogens, including *Bartonella* spp. [7,8,9], *Acinetobacter* spp. [10], *Rickettsia* spp. [10,11], and *Coxiella burnetii* [11]. A study reported by Gutiérrez et al. (2014) demonstrated that *Bartonella* spp. infection in cattle lice; *H. quadripertusus*, and cattle blood from Israel. Moreover, *B. bovis* infection has been reported as a pathogen which causes endocarditis and bacteremia in cattle, and the *B. henselae* infection is the most common cause of cat scratch disease and presents systemic infection and with possible skin lesions in human [12]. The previous studies revealed that *Rickettsia* spp. were detected in *Linognathus* spp. and *H. eurysternus* from ruminants in Hungary [13,14]. Kumsa et al. (2012) showed that different *Acinetobacter* spp. could be found in *L. vituli* of cattle from the Oromia Regional State, Ethiopia [10]. *Acinetobacter* spp. in human head lice collected from school children in Thailand was also reported [15]. However, molecular techniques of cattle lice species and their pathogenic bacteria in Thailand have never been investigated. In this study, we demonstrated the use of molecular techniques for cattle lice species identification and detection of the potential bacterial pathogens in cattle louse samples collected from different areas of Thailand. Information obtained from the study provides fundamental data for the epidemiological study as well as the potential of cattle lice as a vector of zoonotic disease in Thailand.

## 2. Materials and Methods

### 2.1. Collection of Lice from Beef Cattle

The study was approved by the animal research ethics committee of Chulalongkorn University Animal Care and Use Protocol (CU-ACUP), Faculty of Medicine, Chulalongkorn University, Bangkok, Thailand (No. 005/2562). A total of 109 cattle lice samples consisted of 98 adults and 11 eggs. The collections were conducted in three different areas of Thailand, including Chiang Rai, Chiang Mai, and Nakhon Ratchasima provinces. Lice were manually collected from the hair tail and around the face of each cow. All specimens were preserved and surface decontaminated in 70% ethanol and transported to the Vector Biology and Vector Borne Disease Research Unit, Department of Parasitology, Faculty of Medicine, Chulalongkorn University. They were then classified to the genus level by morphological feature key [16,17].

### 2.2. DNA Extraction from Cattle Louse

Genomic DNA was extracted from individual cattle louse, as follows. First, the louse was washed once in 1 mL of sterile 1X phosphate-buffer saline (PBS) for 5 min for removing the 70% ethanol, and then an individual cattle louse of each sample was homogenized in 200 µL of lysis buffer G and 20 µL of proteinase K. The genomic DNA was extracted using a DNA extraction kit, Invisorb^®^ spin tissue mini kit (STRATEC molecular GmbH, Berlin, Germany) following the manufacturer’s instructions. Finally, the extracted cattle lice DNA was obtained in 50 µL of elution buffer. The genomic DNA was stored for long term at −20 °C until use in Polymerase Chain Reaction (PCR) amplification.

### 2.3. PCR for Cattle Louse Identification

Conventional PCR was used for amplifying *18S rRNA* of the cattle louse. Degenerate oligonucleotide primers were designed based on *18S rRNA* sequences of the cattle lice obtained from GenBank database (GenBank: KJ522491 for *H. quadripertusus*, GU569180 for *H. tuberculatus*, HM171381 for *H. eurysterunus*, and AY077774 for *Linognathus vituli*) as forward primer 5′-CCGCGAAAGGCTCATTAAATCAG-3′, and the degenerate reverse primer sequences were 5′-CCTKCAATGGATACTCGTTAAATG-3′. The primers were synthesized by Bioneer Oligo Synthesis Report Company (Bioneer Corporation, Daejeon, Korea). PCR reaction was set up in the final volume of 25 µL containing approximately 50 ng/µL of extracted DNA, 10 µM of each primer, 10X *Taq* buffer, 2.5 mM of dNTPs, 2.5 mM of MgCl_2_ and 1 unit of *Taq* DNA polymerase (Thermo scientific, Waltham, MA, USA); double distilled water was as a negative control. PCR were performed under the following thermal cycling conditions: An initial denaturation step of 95 °C for 3 min, followed by 35 cycles of 95 °C for 1 min, 58 °C for 1 min, and 72 °C 1.30 min, with the final step of 72 °C for 7 min (Figure S1). The PCR amplicons were determined by 1.5% agarose gel electrophoresis, stained with ethidium bromide. The specific of PCR product were imaged under ultraviolet light with Quantity One Quantification Analysis Software version 4.5.2 (Gel DocEQ System; Bio-Rad, Hercules, CA, USA).

### 2.4. Detection of Bacterial Pathogens in Cattle Louse DNA

The cattle lice DNA were used to detect bacterial pathogens by using the PCR assay. The PCRs were performed using previously reported primers targeting the *gltA* of *Bartonella* spp. [18], the *rpoB* of *Acinetobacter* spp. [19], and the *gltA* of *Rickettsia* spp. [20]. For the PCR reaction, 5 µl of DNA template was used in a total volume of 25 µL; the reaction mixture contained 10X *Taq* buffer, 2.5 mM of dNTPs, 2.5 mM of MgCl_2_, 10 µM of each primer, and 1 unit of *Taq* DNA polymerase (Thermo scientific, Lithuania, EU). The PCR cycling conditions were as follows: Initial denaturation at 95 °C for 3 min; 35 cycles of 95 °C for 30 s, 57, 60, and 55 °C for *Bartonella* spp., *Acinetobacter* spp., and *Rickettsia* spp., for 30 s respectively, and 72 °C for 1 min; and the final extension at 72 °C for 7 min (Figure S2). Positive and negative controls were included in each experiment. The PCR amplicons were confirmed by gel electrophoresis described above for louse. In order to confirm the species of the *Bartonella* bacteria, all DNA samples were detected by using conventional PCR amplification targeting a 406 bp fragment of RNA polymerase (*rpoB*) gene [21].

### 2.5. DNA Cloning and Sequencing

The positive PCR products were ligated into pGEM-T Easy Vector (Promega, Madison, WI, USA) using T4 DNA ligase. The DNA ligation was transformed into *Escherichia coli* DH5α and screened using the blue-white colony selection system. The suspected positive colonies were cultured, and the plasmid DNA contained with the insert gene was isolated using the Invisorb^®^ Spin Plasmid Mini Kit (STRATEC molecular GmbH, Berlin, Germany) following the manufacturer’s instructions. Sequencing was performed by a commercial service in MACROGEN, Korea using a universal forward T7 primer.

### 2.6. Sequence Analysis and Phylogenetic Tree Construction

The nucleotide sequences were analyzed using the BioEdit Sequence Alignment Editor Version 7.2.5 [22]. The consensus sequences were analyzed by comparison with the nucleotide sequence in the GenBank database using BLAST search (https://blast.ncbi.nlm.nih.gov/Blast.cgi) and all the nucleotide sequences from this study were submitted to the GenBank database. The phylogenetic trees were constructed using the maximum-likelihood method with IQ-TREE on the IQ-TREE web server (http://iqtree.cibiv.univie.ac.at/) with 1000 ultrafast bootstrap replicates. The best-fit model of substitution was found using the auto function on the IQ-TREE web server [23]. The phylogenetic tree was finally viewed and edited with the FigTree v1.4.4 software.

## 3. Results

### 3.1. Morphology and Molecular Identification of Cattle Lice

**Figure 1:**
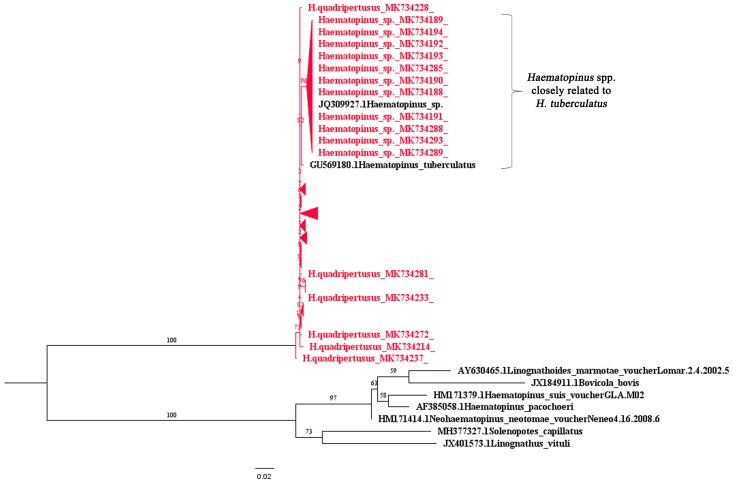
Phylogenetic tree of cattle lice constructed from partial *18S rRNA* sequences. The maximum likelihood was constructed with IQ-TREE by using the maximum-likelihood method with 1000 ultrafast bootstrap replicates. The best-fit model of substitution was found using the auto function on the IQ-TREE web server. The sequences from this study are indicated with a red color.

A total of 109 (11 eggs, 98 adults) cattle lice samples were collected from Chiang Mai (11 eggs, 88 adults), Chiang Rai (7 adults) and Nakhon Ratchasima (3 adults). The morphological characters to identify the species of adult stage cattle lice, showed *H. quadripertusus* (*n* = 95) and *Haematopinus* spp. (*n* = 3); whereas, the species of cattle louse egg was unable to be morphologically identified. The molecular technique, PCR based on the partial *18S rRNA* was developed to identify the cattle louse in Thailand. The portion of *18S rRNA* sequence was 745–746 bp. The results found 98 (7 eggs, 91 adults) samples clustered together with *H. quadripertusus* and 11 (4 eggs, 7 adults) samples of *Haematopinus* spp. closely related to *H. tuberculatus* with short branch lengths, which were identified by the phylogenetic tree base on the *18S rRNA*. (Table 1 and Figure 1). Sequence divergence was 0.2–2.9% between *H. quadripertusus* and *Haematopinus* spp. closely related to *H. tuberculatus* and 0.8–1.5% between *Haematopinus* spp. closely related to *H. tuberculatus* and *H. tuberculatus*. The nucleotide sequences of *18S rRNA* of cattle lice were submitted to the GenBank database, accession numbers MK734185-MK734293 (Table S1).

### 3.2. Detection of Bacterial Pathogen in Eggs and Adults Cattle Louse

**Figure 2:**
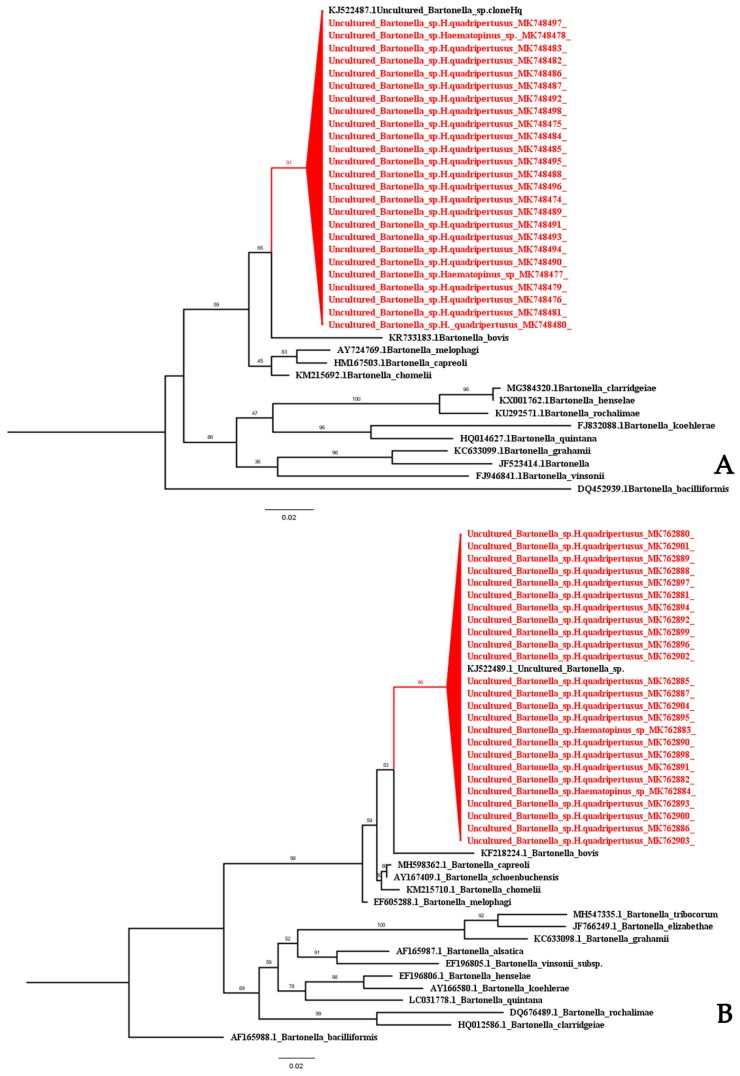
Phylogenetic tree of *Bartonella* spp. from cattle lice based on partial *gltA* (**A**) and *rpoB* (**B**) regions. The maximum likelihood was constructed with IQ-TREE by using the maximum-likelihood method with 1000 ultrafast bootstrap replicates. The best-fit model of substitution was found using the auto function on the IQ-TREE web server. The sequences from this study are indicated with a red color.

In this study, we investigated the PCR of all 109 cattle lice DNA for *Bartonella* spp., *Acinetobacter* spp. and *Rickettsia* spp. Only *Bartonella* spp. DNA was detected in 25 of 109 (22.93%) samples. The positive samples including three egg samples and 22 adult samples. *Bartonella* spp. DNA was detected by PCR targeting the *gltA* in 22 of 98 (22.45%) of *H. quadripertusus* collected from Chiang Mai (2 eggs, 17 adults) and Nakhon Ratchasima (3 adults). The *Haematopinus* spp. closely related to *H. tuberculatus* was detected in three of 11 (27.27%) *Bartonella* spp. DNA from Chiang Mai (one egg) and Chiang Rai (two adults). One hundred and nine of cattle lice were also confirmed by PCR using primers targeting a 406 bp fragment of the *rpoB*. The results showed 25 samples were positive for the *Bartonella* spp. DNA, which were the same samples positive for *gltA* (Table 2). The phylogenetic of *Bartonella* spp. of *gltA* (Figure 2A) and *rpoB* (Figure 2B) from cattle louse were closely related to the *B. bovis*. The partial nucleotide sequence of the *gltA* and *rpoB* obtained in this study was deposited in the GenBank under accession number: MK748474-MK748498 and MK762880-MK762904, respectively (Table S2).

## 4. Discussion

In Thailand, the identification of cattle louse is based on morphological characters. The precise identification depends on various factors such as stage of the louse samples and the experience of entomologist. Adult stages can be identified more accurately by morphology. However, it may lead to misidentification in case of immature stages such as eggs and nymphs of the cattle lice. As previously mentioned, the identification of cattle lice in Thailand has been based only on morphological characters which could be quite problematic. In order to solve this problem, we demonstrated the use of *18S rRNA*-PCR to identify the cattle lice species. To the best of our knowledge, this is the first molecular identification of cattle lice in Thailand. The phylogenetic analysis of *18S rRNA* sequence of cattle louse revealed that *H. quadripertusus* was similar to the *H. quadripertusus* from Israel (Accession no. KJ522491) [12]. The *Haematopinus* spp. in this study showed similar to *Haematopinus* sp. NKU-011 from China (Accession no. JQ309927) [24] and clustered together with *H. tuberculatus* from Japan (Accession no. GU569180) [25]. Thereby, we assumed that the *Haematopinus* spp. in this study were *Haematopinus* spp. closely related to *H. tuberculatus*. The previous studies of domestic cattle and buffaloes lice in Thailand reported that *H. eurysternus*, *H. quadripertusus*, *L. vituli*, and *S. capillatus* were found by using taxonomic identification [5,6]. This preliminary study of phylogenetic tree of 109 cattle louse samples in three regions of Thailand revealed the genetic diversity among the louse samples. However, according to the limitation of information on the molecular evolution as well as sequences data for cattle lice in Thailand, we are not able to compare our results with other studies within the country. Several studies described that both mitochondrial (Cytochrome C oxidase subunit I: *COI*) [26] and nuclear genes (*18S rRNA* and *EF-1α*) [10,26] have been used to study the genetic diversity among cattle lice species. In this present study, the *18S rRNA* was selected because this gene has been previously used as an effective tool to demonstrate the evolution [26] and phylogeny of sucking lice [27]. Moreover, there are *18S rRNA* reference sequences of cattle lice available in GenBank more than other gene regions, which are also used for designing new primers in our study. As a result of this study, data of the *18S rRNA* sequences of the 109 cattle louse samples from Thailand are already deposited in the GenBank.

In order to determine whether pathogenic bacteria could be found in the cattle lice, we performed the PCR assays for *Bartonella* spp., *Acinetobacter* spp., and *Rickettsia* spp. detections. The results showed that *Acinetobacter* spp. and *Rickettsia* spp. DNA were not detected in this study. Interestingly, 25 of 109 *Bartonella* spp. DNA was detected by both primer sets which annealed specially to the *gltA* and *rpoB* genes. *Bartonella* spp. DNA was found in both *H. quadripertusus* and closely related to *H. tuberculatus.* The *gltA* and *rpoB* sequences of *Bartonella* spp. are closely related to uncultured *Bartonella* spp. clone Hq in the cattle tail louse, *H. quadripertusus* Accession no. KJ522487 and KJ522489 from Israeli dairy farms, respectively [12]. In addition, *gltA* and *rpoB* sequences of *Bartonella* spp. closely relate to the *B. bovis* strain I724598 from cattle blood in Malaysia (Accession no. KR733183) [28] and water buffalo blood in Thailand (Accession no. KF218224) [29]. The *Bartonella* bacteria are facultative intracellular bacteria that can be found in a wide range of mammalian and arthropods such as ticks, lice, fleas, and sand flies [30]. Cattle are currently claimed to be reservoirs of three *Bartonella* species including *B. bovis, B. schoenbuchensis*, and *B. chomelii*, however, none of these has been reported as causative agent in humans [31]. There are some reports that the infected arthropods could transmit *Bartonella* bacteria to human and other mammalian hosts such as *B. henselae* from cat fleas (*Ctenocephalides felis*) and *B. quintana* from human body lice (*Pediculus humanus*) [32,33]. The prevalence of *Bartonella* spp. isolated from a large number of rodents and shrews blood were found in several countries in Southeast Asia including Lao PDR (11.9%), followed by Thailand (11%) and Cambodia (9.6%) [34]. In Thailand, many studies suggested the *Bartonella* spp. were detected in a febrile illness as well as endocarditis in patients and their potential animal reservoirs [35,36]. Bai et al. (2013) revealed that the *Bartonella* spp. was isolated from 10% (4/40) of the healthy cattle and *B. bovis* were cultured in 6.8% (7/103) from water buffaloes blood in Thailand [29]. However, data on cattle associated with the *Bartonella* infection in humans is still limited in Thailand. In the literature, high prevalence of *Bartonella* DNA and genotype diversity have been detected among arthropod vectors around the world. For example, the prevalence of *B. bovis* in cattle has shown variability, with reports from Italy (24.2%) [37], France (59%) [38], and Poland (6.8%) [39]. In the United States, the *B. bovis* infection rates in cattle varied across the regions studied, being as high as 81–96% in California [7], 82.4% in North Carolina [40] and less pronounced in Georgia (47%) [41].

In terms of how these arthropods got infected with the bacterial pathogens remains unknown. Some authors suggested the possibility of either acquired infection from animal reservoirs or environmental contamination [10]. The bacterial pathogens could be transmitted among the arthropods through vertical transmission, mating, co-feeding, and fecal exposure. The finding of *Bartonella* spp. in both eggs and adult cattle lice in our study emphasized the possibility of vertical transmission of bacterial pathogen among these arthropods.

This is the first evidence of the discovery of the *Bartonella* spp. DNA in cattle lice in Thailand and detection of the *Bartonella* spp. DNA in eggs of the lice suggested that the vertical transmission of the bacteria in arthropods may occur. Despite the previous negative relation of cattle-associated *Bartonella* spp. causing diseases in human, there is still the feasibility that these bacteria might play a role in zoonotic infection among humans through the bite of blood-sucking insects in the ranch, or accidental contact with infected animals blood via skin abrasion. Further studies are needed to confirm the aforementioned hypothesis. Other blood-sucking arthropods in dairy farms should be targeted in future studies to clarify the potential role of these arthropods as the vectors in the life cycle of the cattle-associated *Bartonella* spp. Regarding the limitations of the study, the number of collected cattle lice were quite low in some regions of Thailand. This could be due to the increased use of insecticides in modern dairy farms to get rid of the cattle lice in order to keep the cattle healthy and hygienic.

## 5. Conclusions

Our findings demonstrated the first use of molecular techniques for the identification of cattle lice species and also showed that *Bartonella* species can be found in cattle lice collected from three regions of Thailand. Both *H. quadripertusus* and *Haematopinus* spp. closely related to *H. tuberculatus* could be the vectors of *Bartonella* spp. Further studies including extensive surveys and more precise studies of cattle lice covering more areas and larger sample sizes must be performed in order to understand the geographical distribution of cattle lice in the country. Moreover, the *Bartonella* spp. prevalence from cattle lice and cattle blood in other locations in Thailand should be investigated to determine the role of domestic animals as the potential sources for human and animal bartonellosis in Thailand.

## Figures and Tables

**Table 1:** Cattle lice samples collected from three different areas of Thailand.

Provinces	Sample No. (n)		Molecular Identification of Cattle Lice (*18S rRNA*)			
	Egg	Adult	*H. quadripertusus* (n)		*Haematopinus* spp. Closely Related to *H. tuberculatus* (n)	
			Egg	Adult	Egg	Adult
Chiang Mai	11	88	7	88	4	NA
Chiang Rai	NA	7	NA	NA	NA	7
Nakhon Ratchasima	NA	3	NA	3	NA	NA
Total	11	98	7	91	4	7
	109		98		11	

NA: Not available.

**Table 2:** Molecular detection of *Bartonella* spp. DNA of *gltA* and *rpoB* from cattle lice samples collected from three different areas of Thailand.

Provinces	*H. quadripertusus* (n)		*Haematopinus* spp. Closely Related to *H. tuberculatus* (n)	
	Egg	Adult	Egg	Adult
Chiang Mai	2/7	17/88	1/4	NA
Chiang Rai	NA	NA	NA	2/7
Nakhon Ratchasima	NA	3/3	NA	NA
Total	2/7	20/91	1/4	2/7
	25/109			

Number: Positive/Total sample tested; NA: Not available.

## Supplementary Materials

The following are available online at https://www.mdpi.com/2075-4450/10/6/152/s1. Figure S1: *18S rRNA*-PCRs for cattle louse identification; Figure S2: PCRs for *Bartonella* spp. Detection; Table S1: Accession numbers of DNA sequences deposited in GenBank for the *18S rRNA* of cattle lice detected in this study; Table S2: Accession numbers of DNA sequences deposited in GenBank for the *gltA* and *rpoB* of *Bartonella* sp. detected in this study.
