# Supplementary figures and images for: The Prevalence of Bartonella Bacteria in Cattle Lice Collected from Three Provinces of Thailand
Source: Insects. 2019 May 28;10(6):152. doi: 10.3390/insects10060152 (PMC6628184; doi:10.3390/insects10060152)

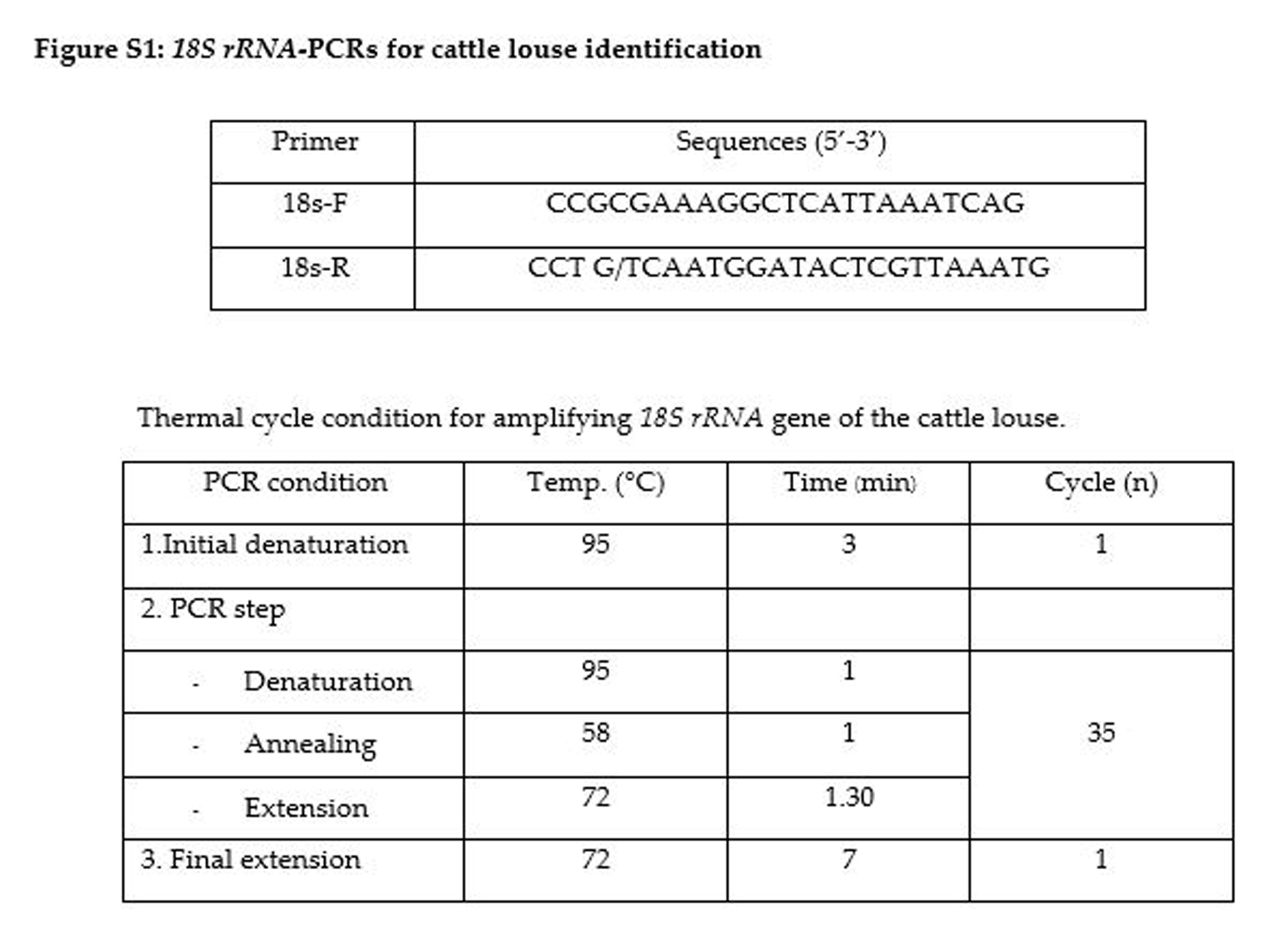

Supplement: Supplementary file 1 [file insects-10-00152-s001.zip › insects-506544-supplymentary/supplement/Figure S1.tif]

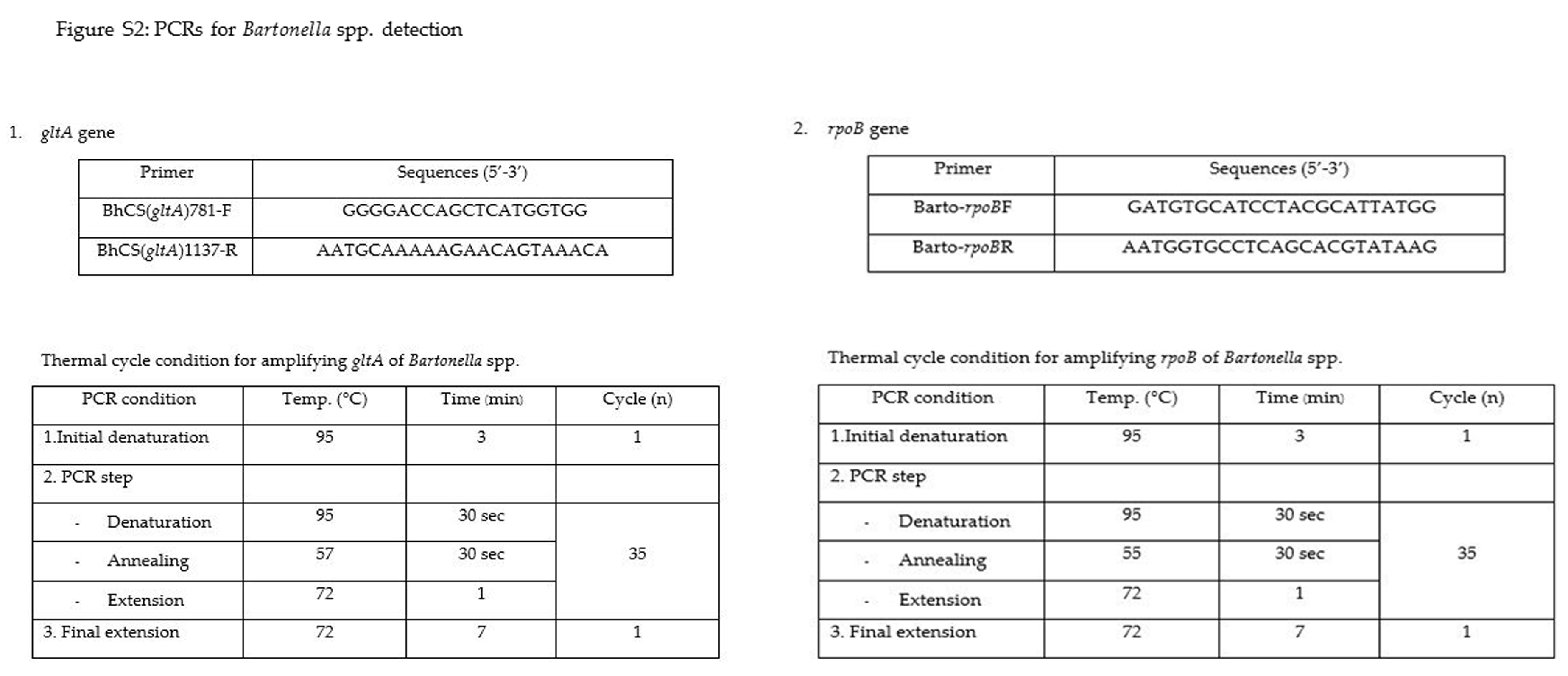

Supplement: Supplementary file 1 [file insects-10-00152-s001.zip › insects-506544-supplymentary/supplement/Figure S2.tif]
